# Prognostic Value of the Fibrosis-4 Index in Human Immunodeficiency Virus Type-1 Infected Patients Initiating Antiretroviral Therapy with or without Hepatitis C Virus

**DOI:** 10.1371/journal.pone.0140877

**Published:** 2015-12-07

**Authors:** Cristina Mussini, Patrizia Lorenzini, Massimo Puoti, Miriam Lichtner, Giuseppe Lapadula, Simona Di Giambenedetto, Andrea Antinori, Giordano Madeddu, Alessandro Cozzi-Lepri, Antonella d’Arminio Monforte, Andrea De Luca, ICONA Foundation study group

**Affiliations:** 1 Clinic of Infectious Diseases, University of Modena and Reggio Emilia, Modena, Italy; 2 National Institute for Infectious Diseases L. Spallanzani, Rome, Italy; 3 Clinic of Infectious Diseases, Maggiore Hospital, Milan, Italy; 4 Clinic of Infectious Diseases, La Sapienza University, Rome, Italy; 5 Department of Infectious Diseases, San Gerardo Hospital, Monza, Italy; 6 Istituto di Clinica delle Malattie Infettive, Università Cattolica del Sacro Cuore, Roma, Italy; 7 Clinica delle Malattie Infettive, Università di Sassari, Sassari, Italy; 8 Department of Infection & Population Health Division of Population Health, Hampstead Campus, University College London, London, United Kingdom; 9 Clinic of Infectious Diseases, Department of Health Sciences, San Paolo Hospital, Milan, Italy; 10 Division of Infectious Diseases, Department of Medical Biotechnologies, University of Siena and Siena University Hospital, Siena, Italy; Harvard Medical School, UNITED STATES

## Abstract

**Objective:**

To evaluate the Fibrosis (FIB)-4 index as a predictor of major liver-related events (LRE) and liver-related death (LRD) in human immunodeficiency virus (HIV) type-1 patients initiating combination antiretroviral therapy (cART).

**Design:**

Retrospective analysis of a prospective cohort study.

**Setting:**

Italian HIV care centers participating to the ICONA Foundation cohort.

**Participants:**

Treatment-naive patients enrolled in ICONA were selected who: initiated cART, had hepatitis C virus (HCV) serology results, were HBsAg negative, had an available FIB-4 index at cART start and during follow up.

**Methods:**

Cox regression models were used to determine the association of FIB4 with the risk of major LRE (gastrointestinal bleeding, ascites, hepatic encephalopathy, hepato-renal syndrome or hepatocellular carcinoma) or LRD.

**Results:**

Three-thousand four-hundred seventy-five patients were enrolled: 73.3% were males, 27.2% HCV seropositive. At baseline (time of cART initiation) their median age was 39 years, had a median CD4+ T cell count of 260 cells/uL, and median HIV RNA 4.9 log copies/mL, 65.9% had a FIB-4 <1.45, 26.4% 1.45–3.25 and 7.7% >3.25. Over a follow up of 18,662 person-years, 41 events were observed: 25 major LRE and 16 LRD (incidence rate, IR, 2.2 per 1,000 PYFU [95% confidence interval, CI 1.6–3.0]). IR was higher in HCV seropositives as compared to negatives (5.9 vs 0.5 per 1,000 PYFU). Higher baseline FIB-4 category as compared to <1.45 (FIB-4 1.45–3.25: HR 3.55, 95% CI 1.09–11.58; FIB-4>3.25: HR 4.25, 1.21–14.92) and time-updated FIB-4 (FIB-4 1.45–3.25: HR 3.40, 1.02–11.40; FIB-4>3.25: HR 21.24, 6.75–66.84) were independently predictive of major LRE/LRD, after adjusting for HIV- and HCV-related variables, alcohol consumption and type of cART.

**Conclusions:**

The FIB-4 index at cART initiation, and its modification over time are risk factors for major LRE or LRD, independently of infection with HCV and could be used to monitor patients on cART.

## Introduction

Combination antiretroviral therapy (cART) had deeply changed the natural history of HIV-associated disorders. In recent years, thanks to increasingly effective cART, the landscape of morbidity and mortality has been characterized by a continuous reduction of AIDS-events and AIDS-related death and a relative increase in non-AIDS events and death by non-AIDS events [1]. Liver-related events (LRE) represent a consistent proportion of non-AIDS events, particularly in patients chronically co-infected with hepatitis viruses, and are a relevant cause of death, particularly in HIV-infected populations with high prevalence rates of HCV [2].

LRE are generated by a chronic hepatic necro-inflammatory damage and its repair, which causes multi-stage progressive fibrosis, leads to cirrhosis and its complications and to hepatocellular carcinoma (HCC). The main underlying causes are HBV or HCV co-infections, alcohol abuse and metabolic disorders with liver involvement. The stage of liver fibrosis can be determined by biopsy or by non-invasive methods, such as transient elastography or measurement of serum markers [3]. The FIB-4 index is one of these non-invasive serum fibrosis markers, which is determined using commonly available parameters such as transaminase levels, platelet counts and age. FIB-4 was initially developed and validated as a predictor of advanced fibrosis in HIV/HCV coinfected patients [4]. Later, It has been validated in HCV mono-infected individuals where it represents a valid predictor of histologic liver fibrosis stage [5] and subsequent LRE and liver-related death (LRD) [6,7], in some cases with better prognostic value than liver biopsy [8], although results are not consistent throughout studies [9].

In HIV/HCV co-infected patients, FIB-4 correlates with liver stiffness as measured by transient elastography and with liver fibrosis score determined by biopsy [10,11]. Moreover, in HIV/HCV co-infected women FIB-4 predicts all-cause mortality [12] and in HIV-infected individuals, this fibrosis index predicts HCC, independently of HCV co-infection [13]. Recently, in HIV/HCV co-infected individuals FIB-4 has also been shown to be an independent predictor of hepatic decompensation [14] as well as to more accurately predict LRE and overall death as compared to liver biopsy [15].

The World Health Organization issued guidelines proposing the systematic use of FIB-4 for the assessment of liver fibrosis in HCV-infected patients and the identification of those who should be given higher treatment priority in resource limited settings [16].

Based on these considerations we analyzed a cohort of HIV-infected HBsAg negative individuals initiating cART, with or without HCV co-infection, in order to assess the predictors of FIB-4 and to evaluate the prognostic value of FIB-4 for major LRE and LRD.

## Patients and Methods

### Patients, FIB-4 index and definition of events

We selected patients from the ICONA cohort, an Italian, national multicenter prospective cohort enrolling adult HIV-infected individuals naïve for antiretroviral therapy [17]. Selection criteria for patients to be included in this study were: to start cART, have an available HCV antibody result, to be HBsAg negative and have an available FIB-4 index at cART start and during follow up. FIB-4 index was calculated by the formula:

$$FIB-4=\frac{Age\;(years)\times AST\;(U/L)}{Platelet\;count\;(10^{9}/L)\times {\left[ALT\;(U/L)\right]}^{1/2}}$$

The resulting values were employed both as continuous variable and divided in categories as follows: FIB-4 value >3.25 as a proxy for advanced fibrosis, consistent with cirrhosis; FIB-4 value between 1.45 and 3.25 in which fibrosis status is considered as undetermined; FIB-4 value<1.45 considered as mild fibrosis or absence of significant fibrosis and consistent with absence of cirrhosis.

Alcohol use was reported by the patient based on non-structured interview of the treating clinician and was classified as daily alcohol intake, occasional alcohol intake, alcohol intake without specification, no alcohol intake or not recorded.

In patients positive for HCV antibodies, HCV RNA was classified as positive or negative based on last available result. Cases without a recorded HCV RNA test result were recorded as HCV RNA not available.

Treatment for HCV was classified as any case treated with interferon, irrespective of the type and associated drugs. During the study period, interferon-free HCV treatment regimens were not available in Italy for this population.

Major LRE were defined by the presence of variceal or gastrointestinal bleeding, ascites, hepatic encephalopathy, other signs of liver de-compensation including hepato-renal syndrome or by a diagnosis of hepatocellular carcinoma (HCC). Deaths were classified as liver-related (LRD) when associated to recent major liver events. Both major LRE and LRD were confirmed by onsite monitoring.

All patients enrolled gave written, informed consent to participation in the ICONA Foundation cohort study and the study was approved by each of the Ethics Committees from the sites participating to the ICONA Foundation Study (see the complete list of the Ethics Committees in the supporting information).

### Statistical methods

Baseline of the analysis was the date of cART initiation (between April 1, 1997 and October 31, 2013) and patients were followed until the date of occurrence of the first major LRE or LRD. The follow-up was censored at last clinical visit or at death for causes other than those liver-related or December 31, 2013, whichever occurred first.

Incidence rates were calculated as number of major LRE and LRD divided by person years of follow-up (PYFU).

Multivariable logistic regression with forward stepwise selection was employed to determine the factors associated with baseline FIB-4 >1.45 and >3.25. The models included co-variates showing a significant association (p<0.05) at univariable analysis. Standard survival analysis with Kaplan-Meier curves was used to determine the association of baseline FIB-4, categorized as specified above, with the time to major LRE and LRD. The risk estimates were compared between categories using the log-rank test. Univariable and multivariable Cox regression was also employed to determine the predictors of time to major LRE and LRD. Variables with a p-value <0.05 at the univariable analysis were included in the multivariable model using a stepwise forward selection method. Both, baseline and time-dependent values were employed for HIV RNA and CD4 values as well as for the FIB-4 category. In a separate multivariable model, the predictive value of the change from baseline to current value of FIB-4, as a continuous variable, was also analysed. The accuracy of a baseline FIB-4 >1.45 and >3.25 in predicting LRE/LRD were also tested by using the area under the receiver operating characteristic (AUROC) curves, sensitivity and specificity.

## Results

### Baseline patients characteristics and factors associated with baseline FIB-4

Three-thousand four-hundred seventy-five patients fulfilled the selection criteria. Their characteristics at cART initiation are summarized in Table 1. The majority were male and were infected via heterosexual contacts, 23% reported injecting drug use; they had been diagnosed with HIV since a median of 1 year, had a median plasma HIV RNA of 4.9 log_10_ copies/mL and CD4 counts of 260 cells/μL. About 17% had a previous AIDS-defining condition and 944 (27.2%) had positive HCV-antibodies. Of these, 484 (51.3%) had a HCV RNA result available with 92.1% showing a detectable viral RNA. While most patients started cART between 1997 and 2000, 34% initiated treatment during the most recent calendar years.

**Table 1:** Characteristics of the study population at ART initiation (n = 3475).

	Overall (N = 3475)	HCV-Ab negative (N = 2531)	HCV-Ab positive (N = 944)	p-value (HCV vs. HCV+)
Male gender, n (%)	2549 (73.3%)	1849 (73.1%)	700 (74.2%)	0.519
Age, median (IQR)	39 (IQR 33–45)	41 (IQR 35–46)	35 (IQR 33–40)	<0.001
Mode of HIV transmission, n (%)				
Heterosexual contacts	1483 (42.7%)	1340 (52.9%)	143 (15.1%)	<0.001
MSM	974 (28.0%)	912 (36.1%)	62 (6.6%)	
IVDU	800 (23.0%)	76 (3.0%)	724 (76.7%)	
Other/unknown	218 (6.3%)	203 (8.0%)	15 (1.6%)	
Time from HIV diagnosis, years, median (IQR)	1.0 (0.1–5.5)	0.3 (0.1–3.0)	6.6 (1.0–12)	<0.001
HIV-RNA, log_10_ copies/mL, median (IQR)	4.9 (4.3–5.4)	4.9 (4.4–5.4)	4.8 (4.2–5.3)	<0.001
CDC C stage, n (%)	576 (16.6%)	430 (17.0%)	146 (15.5%)	0.283
CD4+ T cells/μL, median (IQR)	260 (119–378)	260 (116–376)	260 (122–381)	0.689
Blood glucose mg/dL, median (IQR)	87 (80–94)	87 (79–94)	87 (81–96)	0.130
Total Cholesterol, mg/dL, median (IQR)	158 (134–187)	161 (136–188)	150 (124–179)	<0.001
HDL cholesterol, mg/dL, median (IQR)	38 (31–47)	38 (31–47)	37 (30–47)	0.166
LDL cholesterol, mg/dL, median (IQR)	96 (73–117)	98 (77–118)	83 (57–110)	<0.001
ALT, U/L	29 (IQR 19–48)	26 (IQR 18–39)	46 (IQR 27–76)	<0.001
AST, U/L	28 (21–42)	25 (20–34)	44 (28–68)	<0.001
Platelets, cells x 10^9^/L	185 (144–232)	193 (153–239)	164 (121–209)	<0.001
FIB-4 index				
<1.45	2291 (65.9%)	1862 (73.6%)	429 (45.4%)	<0.001
1.45–3.25	917 (26.4%)	562 (22.2%)	355 (37.6%)	
>3.25	267 (7.7%)	107 (4.2%)	160 (17.0%)	
Alcohol intake at baseline				
No intake	1761 (50.7%)	1368 (54.0%)	393 (41.6%)	<0.001
Daily intake	266 (7.6%)	156 (6.2%)	110 (11.6%)	
Occasional intake	781 (22.5%)	567 (22.4%)	214 (22.7%)	
Intake without specification	102 (2.9%)	71 (2.8%)	31 (3.3%)	
Not recorded	565 (16.3%)	369 (14.6%)	196 (20.8%)	
HCV RNA				
negative			38 (4.0%)	
positive			446 (47.3%)	
not available			460 (48.7%)	

MSM, men having sex with men; IVDU intravenous drug users.

Almost two thirds of patients showed a baseline FIB-4 index <1.45 (consistent with absence of cirrhosis), while 26% had an index between 1.45 and 3.25 (indeterminate) and almost 8% had a FIB-4 > 3.25 (consistent with cirrhosis). As compared to HCV-Ab negative individuals, subjects with a positive HCV serostatus at cART initiation were younger, had more frequently injecting drug use as risk factor, had a longer time since HIV diagnosis, lower HIV RNA and LDL-cholesterol levels and showed higher transaminases, more advanced liver fibrosis based on the FIB-4 index and a higher proportion with recorded daily alcohol intake.

Factors associated with the baseline FIB-4 index > 1.45 and >3.25 are summarized in Table 2. In multivariable analysis, male gender, HCV-Ab positive status independently from HCV RNA results, a higher baseline HIV RNA, a previous diagnosis of diabetes and daily alcohol intake were all independently predictive of a FIB-4 index >1.45, while MSM as compared to heterosexual contacts and those with higher pre-ART CD4 counts had lower odds of showing an altered FIB-4 at cART initiation. The same factors, except gender and HIV load and with the addition of baseline fatty liver disease and of a longer time from HIV diagnosis were independently associated with a FIB-4 >3.25.

**Table 2:** Predictors of baseline FIB-4 > = 1.45 and >3.25. Univariable and multivariable logistic regression.

	FIB-4 > = 1.45						FIB-4 >3.25					
	Crude OR 95%CI		*P*	Adjusted OR 95%CI		*P*	Crude OR 95%CI		*P*	Adjusted OR 95%CI		*P*
Male gender vs female	1.46	1.24–1.72	<0.001	1.64	1.35–2.00	<0.001	1.37	1.01–1.85	0.042	1.25	0.91–1.72	0.175
Mode of HIV transmission											ne	
Heterosexual	1.00			1.00			1.00					
MSM	0.69	0.58–0.83	<0.001	0.65	0.52–0.80	<0.001	0.64	0.43–0.95	0.028			
IVDU	2.56	2.15–3.06	<0.001	0.88	0.65–1.19	0.413	3.35	2.51–4.47	<0.001			
Other/unknown	1.04	0.76–1.41	0.819	0.92	0.66–1.27	0.596	1.06	0.58–1.93	0.859			
CDC stage C vs A/B	1.71	1.43–2.06	<0.001	1.20	0.97–1.49	0.088	1.30	0.95–1.78	0.096		ne	
Time from HIV diagnosis, per 1 year more	1.05	1.04–1.06	<0.001	1.01	1.00–1.03	0.113	1.08	1.06–1.10	<0.001	1.03	1.00–1.05	0.030
CD4 pre ART (cells/μL)												
0–199	1.00			1.00			1.00			1.00		
200–349	0.56	0.48–0.67	<0.001	0.62	0.50–0.75	<0.001	0.58	0.43–0.78	<0.001	0.53	0.38–0.74	<0.001
> = 350	0.33	0.27–0.40	<0.001	0.36	0.29–0.44	<0.001	0.37	0.26–0.52	<0.001	0.37	0.25–0.54	<0.001
unknown	0.64	0.46–0.88	0.006	0.65	0.39–1.06	0.082	0.80	0.47–1.35	0.399	0.55	0.25–1.19	0.128
HIV-RNA pre ART (log_10_ copies/mL)												
<4	1.00			1.00			1.00			1.00		
4–4.99	1.17	0.93–1.48	0.175	1.12	0.87–1.44	0.387	1.15	0.76–1.75	0.511	1.12	0.72–1.76	0.612
> = 5	1.66	1.32–2.08	<0.001	1.42	1.10–1.84	0.007	1.25	0.83–1.89	0.281	1.11	0.71–1.74	0.645
unknown	1.56	1.13–2.13	0.006	1.59	1.00–2.55	0.051	1.72	1.01–2.93	0.044	2.37	1.14–4.95	0.021
HCV serostatus												
HCVAb-	1.00			1.00			1.00			1.00		
HCVAb+ HCVRNA-	2.78	1.46–5.29	<0.001	3.10	1.54–6.21	0.001	3.43	1.31–8.97	0,012	2.87	1.03–7.99	0.044
HCVAb+ HCVRNA+	3.42	2.78–4.21	<0.001	3.27	2.42–4.42	<0.001	5.03	3.69–6.85	0,000	4.30	3.00–6.18	<0.001
HCVAb+ HCVRNA na	3.31	2.70–4.06	<0.001	3.32	2.44–4.50	<0.001	4.34	3.17–5.95	0,000	4.57	3.16–6.62	<0.001
Alcohol consumption												
No intake	1.00			1.00			1.00			1.00		
Daily intake	2.20	1.70–2.86	<0.001	1.71	1.29–2.29	<0.001	2.53	1.75–3.66	<0.001	1.83	1,22–2.74	0.003
Occasional intake	0.93	0.78–1.12	0.463	0.83	0.68–1.02	0.071	0.73	0.51–1.04	0.079	0.63	0,44–0.92	0.018
Intake without specification	1.80	1.20–2.69	0.004	1.44	0.93–2.23	0.102	2.73	1.59–4.69	<0.001	2.21	1,23–3.97	0.008
Not recorded	1.10	0.90–1.35	0.346	0.87	0.69–1.10	0.247	0.84	0.57–1.24	0.385	0.66	0,43–1.01	0.055
DM diagnosis before starting cART	2.30	1.31–4.03	0.004	2.40	1.28–4.51	0.007	2.69	1.30–5.61	0.008	4.22	1.93–9.22	<0.001
Total cholesterol at baseline, mg/dL												
< 200	1.00			1.00			1.00			1.00		
> = 200	0.84	0.66–1.06	0.149	0.96	0.74–1.25	0.773	0.54	0.33–0.89	0.015	0.64	0.38–1.10	0.105
Fatty liver disease at baseline	4.47	1.83–10.89	0.001	2.30	0.90–5.90	0.083	5.37	2.19–13.17	<0.001	2.72	1.04–7.10	0.040

MSM, men having sex with men; IVDU intravenous drug users; Na, not available; DM, type 2 diabetes mellitus; Ne not entered, rejected by the forward conditional procedure.

One hundred thirty seven of 944 (14.5%) anti-HCV positive patients were exposed to HCV therapy at any time during the follow up. Of those with a last recorded HCV RNA negative, 8 of 38 (21.1%) were exposed to HCV therapy; of those with last recorded HCV RNA positive, 117 of 446 (26.2%) has been exposed to HCV therapy, while only 12 of 460 (2.6%) without an available HCV RNA result had ever received a treatment for HCV.

### Major liver events and liver-related death

After a median follow-up of 4.1 years (IQR 1.5–8.2), resulting in a cumulative 18,662 PYFU, there were 41 first major LRE and LRD with an incidence rate of 2.2 per 1,000 PYFU (95%CI 1.6–3.0). In detail, there were 25 major liver events (15 had ascites, 5 hepatic encephalopathy, 2 gastrointestinal bleeding, 2 HCC and 1 hepato-renal syndrome), and 16 LRD (“related to liver disease” in 9 cases, “due to complication of chronic viral hepatitis—HCV” in 3 cases, and “due to complication of chronic viral hepatitis—HCV with cirrhosis” in 3 cases and “HCV with liver failure” in 1 case). In the subgroup of 2,531 HCV seronegative individuals there were 6 events over 12,770 PYFU, with an incidence rate of 0.5 per 1,000 PYFU (95%CI 0.2–1.0), while in the subgroup of 944 HCV antibody positive individuals, 35 events were observed over 5,892 PYFU, with an incidence rate of 5.9 per 1,000 PYFU (95%CI 4.3–8.3). There were no major differences in the incidence rates when comparing HCV-Ab positive patients with detectable serum HCV RNA (18 events over 3,528 PYFU, IR 5.1 per 1,000 PY 95%CI 3.2–8.1) with HCV-Ab positive patients without an available HCV RNA (17 events over 2,135 PYFU, IR 8.0 per 1,000 PY 95%CI 4.9–12.8), while no event was observed in HCV-Ab positive HCV RNA negative subjects (0 events over 228 PYFU).

### Predictors of major liver events or liver-related death

**Fig 1:**
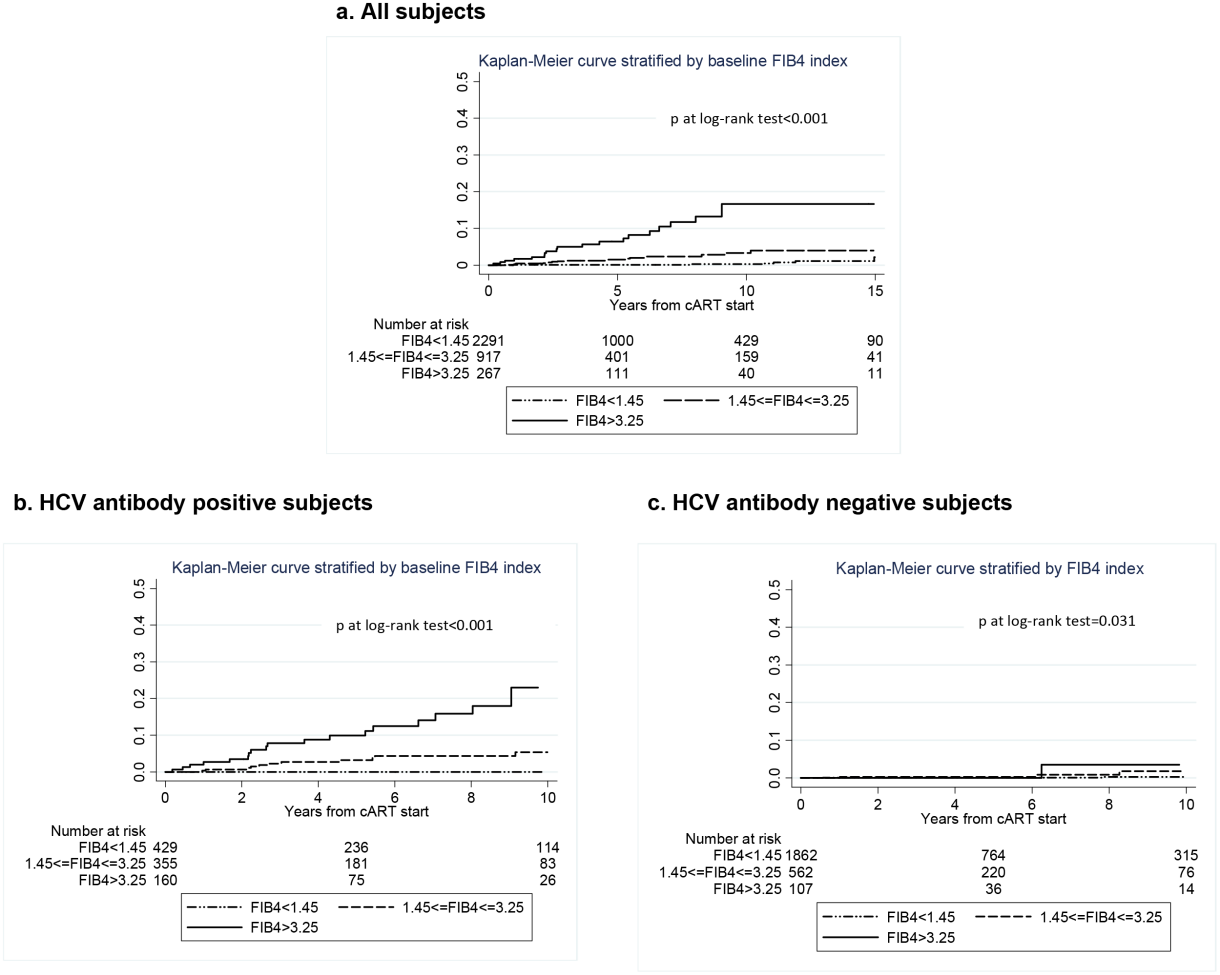
Kaplan-Meier curves showing the estimated probability of major liver events or liver-related death in HIV-infected individuals after cART initiation, according to baseline FIB-4 index category: panel (a) in the whole study population, 41 events in 3475 patients, overall log-rank between categories p<0.001; (b) in the HCV antibody positive population, 35 events in 944 subjects (overall log-rank p<0.001) and (c) in the HCV antibody negative population, 6 events in 2531 individuals (overall log-rank p = 0.031). Dotted line = baseline FIB-4 <1.45, dashed line baseline FIB-4 ≥1.45 and ≤3.25, continuous line = baseline FIB-4 >3.25. Y-axis indicated the proportion of patients with major liver events or liver-related death.

The association between baseline FIB-4 index category and time to a major LRE or LRD is illustrated in Fig 1a. There were 6 events over 12,406 PYFU in the FIB-4 index < 1.45 stratum (incidence rate 0.5 per 1000 PYFU; 95% CI 0.2–1.0), 15 events over 4,885 PYFU in the FIB-4 1.45–3.25 stratum (incidence rate 3.1 per 1,000 PYFU; 1.8–5.1) and 20 events over 1,370 PYFU in those with a FIB-4 index >3,25 (incidence rate 14.6 per 1,000 PYFU; 9.4–22.6). The estimated risk of a major LRE/LRD at 5 and 10 years after cART initiation were 7% and 17%, respectively, in patients with baseline FIB-4 >3.25 while it was lower than 1% at both time points in those with a baseline FIB-4 <1.45. The same association is shown for the subgroups of patients with positive HCV antibodies (Fig 1b) and negative HCV antibodies (Fig 1c). While the association of baseline FIB4 category was evident and significant in the whole population as well as in the HCV-antibody positive subgroup (p<0.001), the HCV-antibody negative subgroup showed a less evident association although statistically significant (p = 0.031), probably due to the small number of events observed in this sub-population. By Univariable Cox regression, factors significantly associated with progression towards major LRE or LRD were injecting drug use, longer time from HIV diagnosis, HCV positive serostatus, baseline and current FIB-4 category, higher score change between current FIB4 and baseline FIB4, alcohol consumption, higher current HIV RNA load and lower current CD4 counts and selected first-line cART types (see Table 3). Multivariable analysis (see Table 3) adjusting for a number of potential confounders, including HCV serostatus, alcohol use, current CD4 and HIV RNA and type of cART, confirmed that both baseline and current FIB-4 index category were strong, independent predictors of major LRE or LRD. In a separate model adjusted for the same co-variates as in the main model except time-updated FIB-4 category, the change from baseline to time-updated FIB-4 index, as a continuous co-variate, also showed an independent association with major LRE/LRD (HR per unit higher 1.03, 95% CI 1.01–1.05, p = 0.001). In a further sensitivity analysis, the same multivariable model was employed on the subset of patients (n = 2,922) with >1 year of follow up. Both, baseline FIB-4 (as compared to <1.45 FIB-4 1.45–3.25: HR 3.30, 95% CI 1.00–10.90; FIB-4>3.25: HR 3.62, 1.00–13.07) and time-updated FIB-4 (FIB-4 1.45–3.25: HR 4.64, 1.27–16.91; FIB-4>3.25: HR 25.24, 7.16–16.22) confirmed an independent association with time to LRE/LRD. Moreover, we tested the accuracy of two baseline FIB-4 index thresholds to predict subsequent LRE/LRD using AUROC curves. A baseline FIB-4 >3.25 showed an AUROC = 0.708 (95%CI 0.692–0.723) with a sensitivity of 48.8% and a specificity of 92.8%, while a FIB-4 >1.45 showed an AUROC = 0.759 (95%CI 0.745–0.774) with a sensitivity of 85.4% and a specificity of 66.5%. Of note, FIB-4 index and HCV-antibody status did not show a significant interaction (p = 0.120), indicating that the association between FIB-4 index category and the risk of major LRE or LRD did not vary significantly between HCV-antibody positive and negative individuals.

**Table 3:** Predictors of time to major liver events or liver-related death (n = 41): univariable and multivariable Cox regression analysis.

	Univariable analysis				Multivariable analysis			
	HR	95% CI		*P*	HR	95% CI		*P*
Male gender vs female	1.25	0.61	2.55	0.542	nt			
Transmission mode								
Heterosexual contacts	1.00				ne			
MSM	0.34	0.04	2.92	0.326				
IVDU	10.95	4.28	28.03	<0.001				
Other/unknown	1.44	0.17	12.34	0.739				
Time from HIV diagnosis (+1 yr more)	1.13	1.08	1.18	<0.001	Ne			
CD4 per cART (cells/μL)					Nt			
0–199	1.00							
200–349	1.44	0.67	3.12	0.350				
>350	1.40	0.65	3.03	0.390				
HIV RNA pre ART (log_10_ copies/mL)					Nt			
<4	1.00							
4–4.99	1.60	0.61	4.23	0.342				
> = 5	0.80	0.28	2.28	0.678				
CDC stage C vs A/B	0.66	0.26	1.68	0.381	Nt			
HCV Ab and HCVRNA								
HCVAb-	1.00				1.00			
HCVAb+ HCVRNA-	-				-			
HCVAb+ HCVRNA+	10.71	4.23	27.08	<0.001	2.79	0.84	9.24	0.093
HCVAb+ HCVRNA na	17.12	6.75	43.43	<0.001	4.43	1.32	14.85	0.016
FIB-4 at baseline								
<1.45	1.00				1.00			
1.45–3.25	6.39	2.48	16.48	<0.001	3.55	1.09	11.58	0.035
>3.25	30.62	12.29	76.28	<0.001	4.25	1.21	14.92	0.024
Score difference between current fib4 and baseline fib4 (+1 point higher)	1.02	1.00	1.08	0.013	Nt			
Current FIB-4								
<1.45	1.00				1.00			
1.45–3.25	7.02	2.36	20.90	<0.001	3.40	1.02	11.40	0.047
>3.25	95.23	39.15	231.65	<0.001	21.24	6.75	66.84	<0.001
Alcohol consumption								
No intake	1.00				1.00			
Daily intake	3.81	1.56	9.27	0.003	1.69	0.62	4.57	0.302
Occasional intake	0.91	0.36	2.33	0.845	0.91	0.33	2.48	0.857
Intake without specification	10.94	4.67	25.62	<0.001	6.36	2.13	18.97	0.001
Not recorded	1.63	0.54	4.94	0.383	0.60	0.12	2.93	0.531
Blood glucose at baseline								
<126 mg/dL	1.00				1.00			
> = 126 mg/dL	4.20	0.97	18.20	0.055	1.82	0.35	9.39	0.476
Not measured	1.65	0.86	3.13	0.129	2.42	0.93	6.31	0.070
ART type NRTI								
TDF+FTC	1.00				1.00			
ZDV+3TC	2.06	0.58	7.30	0.260	1.92	0.40	9.15	0.413
d4T+3TC	3.53	0.85	14.62	0.082	1.94	0.34	11.23	0.457
d4T+ddI	5.07	1.29	19.94	0.020	2.71	0.49	15.02	0.255
other	1.37	0.33	5.64	0.664	0.41	0.06	2.81	0.361
ART type 3^rd^ drug								
NNRTI	1.00				1.00			
bPI	0.46	0.15	1.41	0.174	0.56	0.14	2.14	0.393
uPI	0.89	0.44	1.81	0.746	0.35	0.14	0.90	0.029
NRTI	1.37	0.31	6.08	0.678	5.35	0.73	39.45	0.100
other	3.28	0.74	14.59	0.119	5.13	0.87	30.21	0.071
Current CD4 (+100 cells/μL higher)	0.69	0.59	0.80	<0.001	0.84	0.71	1.00	0.058
Current HIV-RNA (+1 log_10_ cp/mL higher)	1.56	1.24	1.96	<0.001	Ne			

MSM, men having sex with men; IVDU intravenous drug users; Na, not available; NRTI, nucleos(t)ide reverse transcriptase inhibitors; NNRTI non-nucleoside reverse transcriptase inhibitors; uPI, unboosted protease inhibitors; bPI, boosted protease inhibitors; Ne not entered, rejected by the forward conditional procedure; nt, not tested.

## Discussion

In this study we first analyzed the factors associated with altered pre-cART FIB-4, a noninvasive serum marker of liver fibrosis available for virtually all HIV-1 infected patients. In an adjusted analysis we showed that, as expected, HCV-Ab positive serostatus was an independent predictor of altered FIB-4. All categories of HCV RNA (negative, positive, undetermined) resulted independently associated with more advanced FIB-4 index, suggesting that active HCV replication but also co-factors associated with HCV exposure may play a role in determining more advanced liver fibrosis in HIV.

The independent association of diabetes mellitus and of fatty liver disease with higher FIB-4 index highlights the role of the metabolic syndrome in the progression of liver fibrosis in this HIV-infected population. Independently from that, daily alcohol intake was also a predictor of more advanced FIB-4, which is in agreement with previous observations establishing a strong link between alcohol use and FIB-4 index, particularly in the setting of HIV/HCV co-infection [18]. In addition to this, HIV-specific markers such as high pre-ART viral load and lower CD4 counts were independently associated with a higher FIB-4 index, suggesting that HIV disease progression may favor the progression of liver fibrosis per se. Interestingly, a gradient of reduced risk of liver fibrosis was observed with increased CD4 counts strata, in particular the lowest risk was observed for patients with CD4 counts higher than 350 cells/microliter. These results confirm and substantiate previous observations suggesting a potential relationship between HIV infection and hepatic fibrosis in vivo [19] and underscore the importance of an early ART initiation, with the additional goal to prevent progression of liver fibrosis, independently from viral hepatitis co-infections.

We then showed that pre-ART FIB-4 index had a strong and independent association with progression to major LRE or LRD. The prognostic value of this marker had been previously established in the HCV-infected population, both mono-infected and co-infected with HIV [14]. Sensitivity, specificity and AUROC of a baseline FIB-4 >3.25 in predicting LRE/LRD were very similar to those previously reported in a cohort of HIV/HCV co-infected patients [15]. Of note, using the baseline FIB-4 >1.45 threshold, the accuracy of the prediction increased, due to a significant increase of sensitivity that occurred however at the price specificity. Here we demonstrate that the association of FIB-4 with LRE/LRD in the HIV-infected population, is independent from HCV co-infection status. In fact, although the association was much stronger in the HIV/HCV co-infected group, there was an association also in HCV seronegative individuals and multivariable analysis confirmed an independent association between baseline FIB-4 and LRE/LRD also after adjusting for HCV infection status. This is in agreement with a previous study showing an association of FIB-4 with the development of HCC in HIV-infected individuals, independently from HCV co-infection [13]. Since in the present study patients with positive HBsAg were excluded from the analysis, and relative hazards were adjusted for alcohol use, we hypothesize that the observed association might due to metabolic disorders causing liver fibrosis and its progression, such as non-alcoholic fatty liver disease (NAFLD) [20–22], as well as by other unmeasured variables, such as exogenous toxic causes, or possibly by HIV infection itself [23]. However, we were unable to demonstrate a role of NAFLD in determining LRE/LRD, in part because this cause was presumably underdiagnosed in our cohort (only 23 patients had a baseline diagnosis of fatty liver disease). In addition, despite diabetes was strongly associated with a higher FIB-4 index, it was not associated with progression to LRE/LRD (not shown) and a higher pre-ART glucose level showed a weak association at the univariable but not at the adjusted analysis. In patients with neither HCV infection nor alcohol abuse, the number of LRE/LRD was too small to detect any association (not shown). Moreover, it should be considered that alcohol use was self-reported in this study and, therefore, it could have been underestimated in the analyses. If this was the case, one could expect a more relevant role of alcohol than that already detected in our analyses.

An additional finding was the independent association with major LRE and LRD shown by the change from baseline to current FIB-4 as well as by the time-updated FIB-4 index category. Interestingly, this was not only independent from HCV serostatus but also from baseline FIB-4. This is in agreement with similar observations made in the HCV-monoinfected population [6]. Previous studies have related the evolution of FIB-4 in HIV-monoinfected individuals to uncontrolled HIV viral load and low pre-cART CD4 counts as well as to the use of ddX drugs [23] and in patients with HIV and hepatitis virus coinfections, modification in the fibrosis score was associated with all-cause mortality [24]. Importantly, in our analysis we found no interaction between baseline FIB-4 index category and HCV-antibody status in predicting the progression to major LRE/LRD. These findings suggest the requirement of a monitoring of this serum marker over time in the HIV-infected population, independently from HCV serostatus. Altogether these observations on the prognostic value of baseline and time-updated FIB-4 in HIV suggest that this as a relevant marker that can be employed for prospective interventional studies both in the HCV co-infected population, where it might be used to prioritize the newer treatment options with directly acting antivirals, as well as in the HCV negatives where it might be used for other specific interventions aimed at reducing the risk of fibrosis evolution, by acting on modifiable factors such as the control of diabetes and, in particular, stopping alcohol use and increasing the CD4 counts, which may best be obtained by an early ART initiation [23,25].

This study has several limitations. First, HCV RNA measurement was available in only part of the HCV-Ab positive patients. Hence, the effect of active HCV replication was documented only for one half of the HCV-exposed population, which reduced our power to detect the role of active HCV replication in inducing progression of liver disease. However, since 92% of those who were HCV seropositive and were tested had a detectable HCV viral load, it is likely that our results are sufficiently robust, as indicated by the similar hazard ratio of LRE/LRD for both groups of HCV RNA positive patients and those without available HCV RNA. Moreover, the role of HCV treatment could not be established, since, as in other cohort studies [26,27], the proportion of treated patients was limited, it was based on interferon plus ribavirin in most cases (not shown), and the expected success rate was poor. The smallest rate of exposure to HCV therapy observed in the group of anti-HCV positive subjects without an HCV RNA documentation suggests that this category of individuals may represent a subset that, for different reasons, including epidemiological factors or co-morbidities, were rarely deemed to be candidate to interferon-based HCV treatment. The same factors may be an explanation for the higher rates of major LRE/LRD observed in this category as compared to those with a documented positive HCV RNA. In particular, during the initial years of antiretroviral treatment the clinicians were more concerned about HIV progression than HCV co-infection; further, anti HCV drugs, in particular interferon, were not indicated in patients with low CD4 counts and this could explain the low number of treated individuals.

Another limitation of this study is the lack of a structured measurement of patients alcohol intake at the source level, which did not allow a more refined exploration of its role in the progression of liver fibrosis and in influencing liver disease outcome, although a structured reporting by the provider allowed some level of insight into alcohol use in our population. In addition, the diagnosis of fatty liver disease was not collected in a standardized fashion and was therefore largely underestimated in the cohort. Lastly, we could not estimate the Child-Pugh and Meld score for patients with cirrhosis; however, our study relied on hard liver-related events and liver-related death episodes, similarly as several other cohort studies.

In conclusion, our study shows that in a cohort of HIV-positive individuals, FIB-4 at cART initiation and its modification thereafter are risk factors for major liver events or liver-associated death, independently of infection with HCV. FIB-4 index represents a relevant marker for monitoring HIV-infected individuals and may be used to prioritize treatment of HCV in this population.
